# Asymmetric Glaucoma and Corresponding Hearing Impairment

**DOI:** 10.3390/jcm13216501

**Published:** 2024-10-30

**Authors:** Laura Antonia Meliante, Giulia Coco, Beatrice Francavilla, Matilde Bianchi, Gianluca Velletrani, Stefano Di Girolamo, Gianluca Manni

**Affiliations:** 1 Department of Clinical Sciences and Translational Medicine, University of Rome Tor Vergata, 00133 Rome, Italy; 2 Department of Otorhinolaryngology, University of Rome “Tor Vergata”, 00133 Rome, Italy; 3 IRCCS—Fondazione Bietti, 00184 Rome, Italy

**Keywords:** glaucoma, unilateral glaucoma, asymmetric glaucoma, hearing loss, hearing impairment

## Abstract

**Background/Objectives**: This study aims to explore the potential relationship between unilateral or asymmetric glaucoma and ipsilateral hearing impairment. **Methods**: In this retrospective study, visual and hearing functions were assessed in patients with unilateral or asymmetric glaucoma. Correlations between retinal nerve fiber layer (RNFL) thickness, visual field mean deviation (MD) values, and pure tone audiometry (PTA) measurements across various frequencies were analyzed to explore potential associations between visual and ipsilateral hearing functions. Differences in PTA values between ears ipsilateral to the more affected glaucomatous eyes and the contralateral ears were studied for statistical significance. **Results**: Twenty-six patients with unilateral or asymmetric glaucoma were included in the study. Significant differences in hearing thresholds between the ears corresponding to the more severely glaucomatous eyes and the contralateral ears were found at 0.7, 1, 1.5, and 3 kHz (*p* < 0.05). Additionally, a statistically significant difference was observed in the speech frequencies (0.5, 0.7, 1, 1.5, 2, 3, and 4 kHz) between ears corresponding to glaucomatous or more affected glaucomatous eyes and the contralateral ears (*p* = 0.016). Furthermore, a moderately positive correlation was found between differences in MD and PTA values at 0.125 kHz (r = 0.50; *p* = 0.01). **Conclusions**: This study highlights a potential association between unilateral or asymmetric glaucoma and ipsilateral hearing impairment, particularly at speech-relevant frequencies. These findings underscore the importance of integrated sensory assessment in the management of glaucoma patients, suggesting that early detection and intervention for concurrent hearing loss could enhance overall quality of life.

## 1. Introduction

Glaucoma, a leading cause of irreversible blindness worldwide, primarily affects the optic nerve, leading to progressive vision loss [1]. A few studies and reviews have examined the potential association of glaucoma and hearing impairment [2]. This relationship could be explained by several potential mechanisms. Glaucoma and hearing loss share common pathogenic factors, including vascular impairment and neurodegeneration [2]. Glaucoma is characterized by the degeneration of the optic nerve, the thinning of retinal nerve fibers, and the loss of retinal ganglion cells, frequently associated with vascular abnormalities such as diminished perfusion pressure and impaired vascular autoregulation [3,4,5,6,7]. Similarly, sensorineural hearing loss (SNHL) results from cochlear and auditory nerve damage, influenced by microvascular alterations causing ischemic injury to inner ear structures [8,9].

Understanding the broader implications of glaucoma is crucial for developing comprehensive management strategies that address all aspects of patients’ quality of life (QoL). Although most affected patients do not report any specific symptoms or vision loss initially, glaucoma can adversely affect patients’ QoL and ability to perform visually related activities, even when they are unaware of their diagnosis [10].

Hearing loss (HL), like vision loss, is a major public health concern that significantly affects daily functioning and social interactions. Effective communication relies heavily on auditory perception, and any impairment can lead to social isolation, depression, and diminished overall well-being [10]. Unlike vision impairment, which primarily affects spatial navigation and interaction with the physical environment, hearing loss hinders social engagement and the ability to participate in conversations [10]. When both vision and hearing impairments co-occur, the negative impact on physical and mental health is compounded, leading to a greater overall decline in QoL than when either impairment occurs alone [10].

Previous studies have identified associations between glaucoma and various forms of HL, particularly in conditions such as pseudoexfoliation syndrome. However, the relationship between primary open-angle glaucoma, pigmentary glaucoma, and auditory deficits has received limited attention [2]. The potential association between unilateral glaucoma and ipsilateral HL remains underexplored. Additionally, the correlations between retinal nerve fiber layer (RNFL) thickness and pure tone audiometry (PTA) values have not been thoroughly investigated. Exploring these relationships is essential for understanding whether the ear ipsilateral to the glaucomatous eye or the one affected by more severe glaucoma exhibits poorer hearing, and for elucidating the potential connections between ophthalmic and auditory health.

This study aims to fill these gaps by evaluating the potential relationship between unilateral or asymmetric glaucoma and hearing impairment ipsilateral to the more affected eye. Additionally, we aimed to investigate whether the presence of glaucoma more noticeably affects auditory performance at speech-relevant frequencies, potentially further impacting patients’ QoL.

## 2. Materials and Methods

In this retrospective study, visual and hearing functions were assessed in patients followed in the Glaucoma Unit at Policlinico Tor Vergata in Rome.

### 2.1. Inclusion and Exclusion Criteria

Patients were included if they had either a diagnosis of unilateral glaucoma or a strongly asymmetric glaucoma. Unilateral glaucoma was defined by the presence in only one eye of (i) glaucomatous defects in the Humphrey 24-2, 30-2, or 10-2 computerized visual field (VF), defined as three or more contiguous points with *p* < 0.05 or two or more contiguous points with *p* < 0.01, or a difference of 10 dB across the nasal horizontal midline in two or more adjacent points, or mean deviation (MD) worse than −5 dB; (ii) ophthalmoscopic diagnosis of glaucoma based on the detection of a glaucomatous optic disc at the fundus examination performed by a glaucoma specialist; (iii) positive Hood glaucoma report at the optical coherence tomography (OCT) for glaucoma. When both eyes met the diagnostic criteria for glaucoma, strongly asymmetric glaucoma was defined in case of: (i) MD difference ≥ 3 dB between the two eyes; (ii) cup-to-disc ratio (C/D) difference ≥ 0.2 between the two eyes; and (iii) peripapillary retinal nerve fiber layer-global (RNFL-g) thickness difference ≥ 20 μm between the two eyes, measured on OCT.

Additional inclusion criteria were the availability of data regarding VF examination, fundus examination, Hood glaucoma report, and PTA at 0.125, 0.250, 0.500, 0.700, 1, 1.5, 2, 3, 4, 6, and 8 kHz, all performed within one year.

Exclusion criteria were applied for both ophthalmological and ear, nose, and throat (ENT) reasons. Ophthalmological exclusion criteria included the presence of unilateral glaucoma secondary to trauma, surgical procedures, ocular inflammation, and/or infections, and patients with angle-closure glaucoma. ENT exclusion criteria included a history of ear or vestibular disorders, any previous ear surgeries, prolonged occupational exposure to high noise levels, a history of ear trauma or chronic ear infections, and the use of medications known to have ototoxic effects.

### 2.2. Ocular Examination and Glaucoma Diagnosis

All participants underwent full ophthalmological examination by a glaucoma specialist, including best corrected visual acuity (BCVA), Goldman applanation tonometry measurements, as well as anterior slit-lamp biomicroscopy and fundus examination. Visual field assessments were conducted using a Humphrey Field Analyzer HFAII-740i and HFAIII (Carl Zeiss Meditec, Inc., Dublin, CA, USA), employing a 24-2 threshold program with the Swedish Interactive Threshold Algorithm (SITA) standard testing strategy. Optical coherence tomography (OCT) was performed using the Spectralis OCT (Heidelberg Engineering, Heidelberg, Germany) and the Hood glaucoma report was generated.

### 2.3. Otolaryngologic Examination and Audiometric Assessment

Hearing thresholds for both ears were assessed using the pure-tone audiometry (PTA) at the following frequencies: 0.125, 0.250, 0.500, 0.700, 1, 1.5, 2, 3, 4, 6, and 8 kHz. A comprehensive otological evaluation and otoscopy were conducted by an experienced otolaryngologist.

### 2.4. Statistical Analysis

The Shapiro–Wilk test was used to determine the normality of PTA, mean deviation (MD), and RNFL values. Depending on the data distribution, either Pearson’s or Spearman’s correlation coefficients were used to assess the relationship between PTA values at the tested frequencies and RNFL values and PTA and MD values. Correlations of inter-eye differences in MD and RNFL and inter-ear differences in PTA were also analyzed to further explore potential associations. Paired *t*-tests and Wilcoxon’s matched-pair signed-rank tests were used to compare PTA values between ears ipsilateral to the glaucomatous eye, or the most damaged one in asymmetrical cases, and the contralateral ears. All statistical analyses were performed with the software R (version 4.4.0 GUI 1.80 Big Sur ARM build released on 24 April 2024) and a two-sided *p*-value of 0.05 was considered for statistical significance.

The study followed the tenets of the Declaration of Helsinki and was approved by the local ethical committee.

## 3. Results

Twenty-six patients were included in the study. Twenty-three had primary open-angle glaucoma (POAG), two had pigmentary glaucoma, and one had pseudoexfoliative glaucoma (PXG). The mean age was 71.69 ± 9.54 and 18 of the patients were male (69.2%).

All patients had unilateral (n = 21) or asymmetric glaucoma (n = 5), affecting the right eye in 11 cases and the left eye in 15 cases. All 26 patients included in this study were Italian and of Caucasian ethnicity.

### 3.1. Ophthalmological Characteristics

The glaucomatous or more severely glaucomatous eyes and the contralateral eyes showed significant differences in several key parameters, as outlined in Table 1. The mean number of hypotensive drops administered was 1.19 ± 1.06 for the glaucomatous or more severely affected eye and 1.35 ± 1.06 for the less glaucomatous eye. Additionally, the mean number of active pharmaceutical ingredients used was 1.42 ± 1.30 for the more glaucomatous eyes, compared to 1.69 ± 1.43 for the contralateral eyes. In terms of surgical interventions, 12 out of 26 eyes in the more glaucomatous group had undergone trabeculectomy or trabeculectomy combined with phacoemulsification. Among these, 2 patients required revision surgery following trabeculectomy, and 1 patient underwent re-trabeculectomy. In contrast, none of the eyes in the non-glaucomatous or less affected glaucomatous group had undergone any surgical interventions for glaucoma.

### 3.2. Pure Tone Audiometry (PTA)

**Figure 1:**
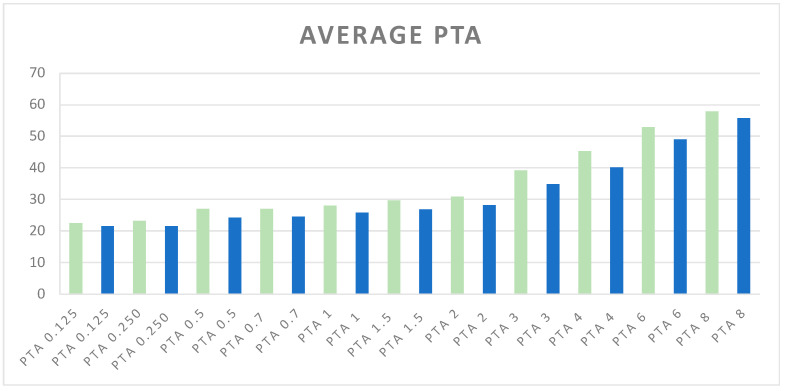
Average of PTA values at different frequencies. Green bars represent PTA values (kHz) for ears corresponding to glaucomatous or more affected glaucomatous eyes, while blue bars represent PTA values for ears corresponding to non-glaucomatous or less affected glaucomatous eyes. The PTA frequencies range from 0.125 kHz to 8 kHz.

The average PTA values, expressed in dB across various frequencies, are illustrated in Figure 1.

These values compare the ears corresponding to glaucomatous or more affected glaucomatous eyes with the contralateral ears, providing a visual representation of hearing thresholds.

### 3.3. Paired t-Tests and Wilcoxon’s Signed-Rank Tests

A Wilcoxon signed-rank test was performed for non-normally distributed data (PTA 0.125, 0.250, 0.7, 1, 2, 3, 6, 8 kHz). Although no statistically significant differences were found at 0.125 kHz or 0.250 kHz (mean differences of 0.96 dB and 1.73 dB, respectively, with small effect sizes of 0.10 and 0.17), statistically significant differences were found at 0.7 kHz (*p* = 0.02), 1 kHz (*p* = 0.03), and 3 kHz (*p* = 0.02); at these frequencies, the mean differences were 2.88 dB, 2.31 dB, and 4.42 dB, respectively, with confidence intervals indicating moderate effect sizes (Cohen’s d = 0.22, 0.18, and 0.20) suggesting that patients with unilateral or asymmetric glaucoma may have hypoacusis in the corresponding ear at these frequencies, thus identifying a potential specific frequency range where glaucoma may impact auditory function. A paired *t*-test was performed for normally distributed data (PTA 0.5, 1.5, 4 kHz). The result of the paired *t*-test at the frequency of 1.5 kHz shows a *p*-value of 0.04, which is statistically significant, with a mean difference of 2.69 dB (95% CI [0.18, 5.20]) and a small effect size (Cohen’s d = 0.18), indicating that hearing thresholds are likely to be affected by glaucoma at this frequency. This result further supports the hypothesis that there may be specific frequencies where glaucoma affects hearing thresholds more noticeably. At higher frequencies, such as 2 kHz and 4 kHz, while there were notable mean differences of 2.69 dB and 5.19 dB, the clinical relevance is less clear due to wider confidence intervals and smaller effect sizes, particularly at 4 kHz (Cohen’s d = 0.23). Similarly, at 6 kHz and 8 kHz, neither the statistical tests nor the effect sizes (Cohen’s d = 0.14 and 0.07, respectively) suggest any significant difference in auditory function between glaucomatous and non-glaucomatous ears. The results of the paired *t*-tests and Wilcoxon’s signed-rank tests, summarized in Table 2, provide detailed insights into the differences in hearing thresholds between glaucomatous and non-glaucomatous ears across various frequencies. The table includes the mean PTA values (±SD) for both groups at each tested frequency, along with the mean differences of PTA between the two groups, 95% confidence intervals, and Cohen’s d as a measure of effect size.

### 3.4. Paired t-Tests for Speech Frequencies

The differences in combined speech frequencies (0.5, 0.7, 1, 1.5, 2, 3, and 4 kHz) between ears corresponding to glaucomatous or more affected glaucomatous eyes and contralateral ears were evaluated [11]. Given that the combined speech frequencies for ears corresponding to non-glaucomatous or less glaucomatous eyes did not follow a normal distribution (Shapiro–Wilk *p* = 0.042), the Wilcoxon signed-rank test was used. The test results of this non-parametric test indicated a statistically significant difference in the combined speech frequencies between the ears corresponding to the glaucomatous or more affected glaucomatous eyes and the contralateral ears (*p* = 0.022). The mean difference in hearing thresholds was 2.40 dB, with a bootstrapped 95% confidence interval ranging from 0.67 to 4.13 dB. Despite the effect size (Cohen’s d = 0.21) indicating a modest clinical impact, these findings imply that glaucoma may still affect auditory function, particularly across these combined speech frequencies. This supports the hypothesis that patients with unilateral or more severe glaucoma could experience worse hearing in the ear corresponding to the glaucomatous or more affected eye.

### 3.5. Correlation Analysis

The correlation analysis between RNFL and MD values for glaucomatous or more affected glaucomatous eyes and PTA of the corresponding ears was performed. The Shapiro–Wilk Test for normality indicated that most PTA values were normally distributed (*p*-value > 0.05), allowing for Pearson’s correlation analysis, except for PTA at 0.125 and 6 kHz frequencies and g-RNFL for glaucomatous eyes (*p*-value ≤ 0.05), thus requiring Spearman’s correlation analysis.

#### 3.5.1. Normality Testing

The Shapiro–Wilk Test for normality indicated that most PTA values were normally distributed (*p*-value > 0.05), allowing for Pearson’s correlation analysis, except for PTA at 0.125 and 6 kHz frequencies and g-RNFL for glaucomatous eyes (*p*-value ≤ 0.05), thus requiring Spearman’s correlation analysis.

#### 3.5.2. RNFL and PTA

Pearson’s correlation coefficients ranged from −0.21 to 0.04, indicating very weak correlations (mostly negative) between PTA values at various frequencies and RNFL. Spearman’s correlation coefficients at non-normally distributed frequencies also showed very weak correlations. None of the *p*-values were below 0.05, indicating that none of these correlations are statistically significant.

#### 3.5.3. MD and PTA

Pearson’s correlation coefficients ranged from −0.19 to −0.38, indicating weak to moderate negative correlations between PTA values at various frequencies and MD of glaucomatous or more affected eyes. The closest to statistical significance was at 4 kHz (*p* = 0.0568).

Although the Pearson and the Spearman correlation analyses between RNFL and PTA values were weak, the negative direction of most correlations suggests a potential trend where thinner RNFL may be associated with worse hearing thresholds. Similarly, the Pearson correlation coefficients between MD values and PTA ranged from −0.12 to −0.38, indicating weak to moderate negative correlations. These negative correlations imply that as the severity of visual field loss (as measured by MD) increases, hearing thresholds tend to worsen. The correlation at 4 kHz was the closest to significance (*p*-value = 0.0568), suggesting a potential trend that warrants further investigation.

#### 3.5.4. Difference Analysis

The results of the correlation analysis are detailed in Table 3. The correlation analysis between the difference in RNFL and MD values for glaucomatous or more affected glaucomatous eyes and the difference in PTA of the corresponding ears was performed. The correlation between differences in MD and PTA at 0.125 kHz showed a moderately positive correlation, with a Pearson’s correlation coefficient equal to 0.5002284 (*p* = 0.009). The correlations generally show that as the difference in PTA between ears corresponding to glaucomatous and non-glaucomatous eyes increases, there tends to be a corresponding increase in the difference in MD values, thus implying that the degree of hearing loss in the ear corresponding to the glaucomatous eye may be related to the severity of visual field loss. However, the strength of these correlations varies across different frequencies, with some frequencies showing moderate positive correlations (e.g., PTA 0.125 and PTA 8), while others show weaker correlations or no correlation at all (e.g., PTA 1, PTA 3, PTA 4). The correlation between differences in RNFL and PTA at 0.125 kHz showed a moderately positive correlation (r = 0.373; *p* = 0.06), indicating that as PTA differences increase, RNFL differences tend to increase moderately, while the correlation between differences in RNFL and PTA at the other frequencies were weak, suggesting that this relationship may be more pronounced at lower frequencies. The results of the difference analysis are detailed in Table 4.

## 4. Discussion

The present study explores the potential link between unilateral or asymmetric glaucoma and ipsilateral hearing impairment, with a specific focus on correlations between RNFL thickness, visual field MD values, and PTA measurements at various frequencies. Our findings underscore the importance of integrated sensory assessments in the comprehensive management of glaucoma patients, given the emerging evidence of a relationship between ophthalmic and auditory health.

In our study, the correlation analysis between MD and PTA values suggested a moderate negative correlation, implying that as the severity of visual field loss increases, hearing thresholds tend to worsen. Furthermore, the correlation between differences in MD and PTA exhibited a moderate positive correlation, indicating that as the difference in PTA between the glaucomatous and non-glaucomatous eyes increases, there tends to be a corresponding increase in the difference in MD values. This finding suggests that the degree of hearing loss in the ear corresponding to the glaucomatous eye may be related to the severity of visual field loss, particularly at lower (0.125 kHz) and higher (8 kHz) frequencies. These findings corroborate the observations made by Neacsu et al., who examined the relationship between MD and PTA and identified an indirect association between visual field examination parameters and audiometry results [12].

The analysis revealed a moderately positive correlation between differences in RNFL thickness and PTA values at the 0.125 kHz frequency. This suggests that as the difference in hearing thresholds between the glaucomatous and non-glaucomatous ears increases, the corresponding difference in RNFL thickness tends to increase moderately. This finding implies that the relationship between ophthalmic and auditory parameters may be more pronounced at lower sound frequencies. This observation corroborates the previous finding that linked the difference in MD to the difference in PTA at 0.125 kHz, further underscoring the importance of examining lower frequencies when investigating the potential connection between glaucoma and hearing impairment.

In this we observed statistically significant differences in speech-relevant frequencies—specifically at 0.7, 1, 1.5, and 3 kHz—between ears corresponding to glaucomatous or more affected glaucomatous eyes and their contralateral ears. This suggests that glaucoma may particularly impact auditory function at these speech-relevant frequencies, which could have profound implications for patient’s quality of life. The paired *t*-tests and Wilcoxon’s signed-rank tests confirmed the statistical significance of these differences, further supporting the hypothesis of a frequency-specific auditory deficit associated with glaucoma. The central, speech-related frequencies appear to be more correlated with glaucoma, potentially because presbycusis typically affects higher frequencies [13]. This age-related HL might reduce the observable difference in hearing thresholds between both sides at higher frequencies. The central frequencies are critical for speech comprehension, and deficits in these ranges can severely impact social interactions and communication. Consequently, the identification of HL at these frequencies underscores the importance of considering hearing rehabilitation options, such as hearing aids, to improve patient’s QoL [14].

Hearing impairments primarily impact social functioning, as hearing is crucial for understanding and participating in conversations, which are central to everyday social interactions. In contrast, vision impairment predominantly affects a person’s ability to navigate and interact with their physical and spatial environment [10]. Research has shown that individuals with both vision and hearing impairments experience more significant declines in physical and mental health, as evidenced by lower scores on health-related quality of life surveys compared to those with only vision or hearing impairment. This compounded effect occurs because vision and hearing influence different aspects of life, and losing both senses results in a greater overall negative impact than losing either one alone [10]. Moreover, glaucoma itself can significantly impact a patient’s QoL in several ways. First, simply receiving a diagnosis of this chronic, irreversible, and potentially blinding disease can cause anxiety and negatively affect well-being [15]. Second, the progressive vision loss associated with glaucoma can lead to difficulties in daily life, affecting activities like reading, driving, and even recognizing faces. This can lead to a loss of independence and social isolation. Finally, the burden of treatment, including the side effects of medications, the inconvenience of drop regimens, and the potential need for surgery, can also negatively impact QoL [15].

The identification of specific frequencies where glaucoma more significantly appears to impact hearing thresholds suggests several broader implications. These findings could influence clinical practices by prompting more comprehensive audiometric evaluations in patients with glaucoma, focusing on the frequencies critical for speech comprehension and communication. This could lead to earlier detection and the management of hearing impairment in this patient population, potentially improving patients’ quality of life through timely interventions such as hearing aids. Furthermore, the pronounced impact of glaucoma on hearing at these speech-relevant frequencies suggests a need to explore potential shared mechanisms or pathways between ocular and auditory health, such as vascular or neurodegenerative processes [6,7,8,16]. Understanding these connections could provide valuable insights into the systemic effects of glaucoma and guide future research and treatment strategies.

The observed association between glaucoma and hearing impairment underscores the need for a holistic approach to patient management. Clinicians should consider routine auditory evaluations in glaucoma patients to identify and address concurrent hearing loss, which can significantly impact patients’ daily functioning and social interactions; a multidisciplinary approach involving ophthalmologists and audiologists could enhance patient care, addressing both aspects of sensory impairment.

The primary limitation of this study was its small sample size. Further research with larger participant groups is necessary to confirm the observed trends and could also help to clarify which specific sound frequencies are most affected by the presence of glaucoma. Another limitation of our study was the lack of assessment of patient’s QoL through the use of specific questionnaires. The retrospective nature of the study precludes causal inferences. Longitudinal studies are necessary to establish the temporal relationship between glaucoma progression and auditory decline. Additionally, larger studies could help elucidate the underlying mechanisms linking visual and hearing impairments in this condition. Although we applied strict inclusion and exclusion criteria, potential confounders such as systemic diseases (e.g., diabetes, hypertension) could influence the results. Future research should aim to control for these variables to isolate the specific impact of glaucoma on hearing function. Future studies should aim to uncover the mechanisms connecting glaucoma with auditory impairment, particularly at the specific frequencies identified in this research. A deeper understanding of these mechanisms could provide valuable insights into the pathophysiology of glaucoma and its broader systemic effects. Exploring the clinical implications of these findings could lead to more comprehensive management strategies, addressing both visual and auditory health for patients with glaucoma.

## 5. Conclusions

This study highlights a potential association between unilateral or asymmetric glaucoma and ipsilateral hearing impairment, particularly at specific speech-relevant frequencies. The findings emphasize the importance of integrated sensory assessments in the management of glaucoma patients, with implications for improving overall QoL through early detection and intervention for concurrent HL. Such research has the potential to lead to improved diagnostic and therapeutic approaches, ultimately enhancing the quality of life for individuals with glaucoma by comprehensively addressing both their visual and auditory needs.

## Figures and Tables

**Table 1:** Mean values of baseline ophthalmological parameters in glaucomatous or more severely glaucomatous eyes and the contralateral eyes.

	More Severely/Glaucomatous Eyes	Less Severely/ Non-Glaucomatous Eyes	*p*-Value
RNFL (micron)	55.88 ± 12.87	93.66 ± 8.93	0.00 ^p^
BCVA (logMAR)	0.17 ± 0.19	0.08 ± 0.18	0.02 ^w^
MD (dB)	−12.8 ± 10.58	−0.38 ± 1.83	0.06 ^p^
IOP (mmHg)	14 ± 6.3	14 ± 3.1	0.45 ^w^

BCVA: best corrected visual acuity; MD: mean deviation; IOP: intraocular pressure; ^p^: paired *t*-test; RNFL: retinal nerve fiber layer thickness; ^w^: Wilcoxon’s signed-rank test.

**Table 2:** Paired *t*-test and Wilcoxon’s signed-rank test results.

PTA Frequency [kHz]	PTA G (Mean ± SD) [dB]	PTA NG (Mean ± SD) [dB]	Mean Difference (PTA G-PTA NG) ± SD [dB]	95% CI	Effect Size (Cohen’s d)	*p*-Value
0.125 ^w^	22.5 ± 9.08	21.54 ± 9.77	0.96 ± 8.25	[−2.37; 4.29]	0.10	0.56
0.250 ^w^	23.27 ± 9.79	21.54 ± 10.93	1.73 ± 7.47	[−1.28; 4.75]	10.37	0.24
0.5 ^p^	27.1 ± 11.85	24.23 ± 11.02	2.88 ± 7.50	[−1.47; 5.91]	11.44	0.06
0.7 ^w^	27.71 ± 13.75	24.58 ± 12.24	2.88 ± 6.02	[0.44; 5.31]	13.03	0.02 *
1 ^w^	28.08 ± 12.66	25.77 ± 13.09	2.31 ± 6.14	[0.23; 4.38]	12.88	0.03 *
1.5 ^p^	29.79 ± 15.29	26.88 ± 14.36	2.69 ± 6.20	[0.18; 5.20]	14.83	0.04 *
2 ^w^	30.96 ± 16.79	28.27 ± 15.29	2.69 ± 6.51	[0.06; 5.32]	16.06	0.06 *
3 ^w^	39.23 ± 23.01	34.81 ± 21.14	4.42 ± 8.64	[0.93; 7.91]	22.08	0.02 *
4 ^p^	45.38 ± 25.57	40.19 ± 20.02	5.19 ± 18.35	[−2.22; 12.60]	23.03	0.16
6 ^w^	52.90 ± 28.36	49.04 ± 27.24	3.85 ± 18.12	[−3.47; 11.17]	27.80	0.31
8 ^w^	57.86 ± 33.11	55.7 ± 29.86	2.11 ± 16.44	[−4.53; 8.76]	31.54	0.49

* *p* < 0.05; ^p^ = paired *t*-test; ^w^ =Wilcoxon signed-rank test; G: more glaucomatous eyes or more affected glaucomatous eyes; NG: non glaucomatous eyes or less affected glaucomatous eyes; CI: confidence interval.

**Table 3:** Correlation analysis between RNFL and PTA and MD and PTA at the studied frequencies.

PTA Frequency (kHz)	RNFL and PTA		MD and PTA	
	Pearson’s Correlation Coefficient	*p*-Value	Pearson’s Correlation Coefficient	*p*-Value
0.125	0.08	0.71	−0.23	0.26
0.250	−0.17	0.40	−0.19	0.34
0.5	−0.12	0.56	−0.22	0.27
0.7	−0.17	0.43	−0.34	0.10
1	−0.11	0.58	−0.30	0.13
1.5	−0.11	0.59	−0.28	0.19
2	−0.21	0.30	−0.34	0.09
3	−0.17	0.40	−0.32	0.10
4	−0.17	0.40	−0.38	0.06
6	−0.02	0.90	−0.32	0.11
8	0.04	0.85	−0.28	0.16

**Table 4:** Correlation analysis between difference in RNFL and difference in PTA; difference in MD and difference in PTA at the studied frequencies. Δ: difference.

PTA Frequency (kHz)	ΔRNFL and ΔPTA		ΔMD and ΔPTA	
	Pearson’s Correlation Coefficient	*p*-Value	Pearson’s Correlation Coefficient	*p*-Value
0.125	0.37	0.06	0.50	0.01
0.250	−0.11	0.59	0.23	0.25
0.5	−0.13	0.54	0.24	0.24
0.7	0.03	0.90	0.27	0.21
1	−0.06	0.77	0.13	0.51
1.5	−0.01	0.96	0.18	0.41
2	0.06	0.76	0.23	0.26
3	−0.36	0.07	-0.03	0.89
4	−0.24	0.24	0.03	0.89
6	−0.27	0.18	0.10	0.64
8	−0.07	0.73	0.30	0.14

## Data Availability

Data supporting the results are available upon request.
